# Prospective monitoring of cefepime in intensive care unit adult patients

**DOI:** 10.1186/cc8941

**Published:** 2010-04-01

**Authors:** Thomas M Chapuis, Eric Giannoni, Paul A Majcherczyk, René Chioléro, Marie-Denise Schaller, Mette M Berger, Saskia Bolay, Laurent A Décosterd, Denis Bugnon, Philippe Moreillon

**Affiliations:** 1 Department of Ambulatory Medicine and Community Healthcare, University of Lausanne, 44, rue du Bugnon, 1011 Lausanne, Switzerland; 2 Department of Pediatrics, CHUV, University of Lausanne, 46, rue du Bugnon, 1011 Lausanne, Switzerland; 3 Department of Fundamental Microbiology, University of Lausanne, Biophore Building, Dorigny, 1015 Lausanne, Switzerland; 4 Department of Adult Intensive Care Medicine and Burns Center, CHUV, University of Lausanne, 46, rue du Bugnon, 1011 Lausanne, Switzerland; 5 Division of Clinical Pharmacology, CHUV, University of Lausanne, 46, rue du Bugnon, 1011 Lausanne, Switzerland

## Abstract

**Introduction:**

Cefepime has been associated with a greater risk of mortality than other beta-lactams in patients treated for severe sepsis. Hypotheses for this failure include possible hidden side-effects (for example, neurological) or inappropriate pharmacokinetic/pharmacodynamic (PK/PD) parameters for bacteria with cefepime minimal inhibitory concentrations (MIC) at the highest limits of susceptibility (8 mg/l) or intermediate-resistance (16 mg/l) for pathogens such as *Enterobacteriaceae*, *Pseudomonas aeruginosa *and *Staphylococcus aureus*. We examined these issues in a prospective non-interventional study of 21 consecutive intensive care unit (ICU) adult patients treated with cefepime for nosocomial pneumonia.

**Methods:**

Patients (median age 55.1 years, range 21.8 to 81.2) received intravenous cefepime at 2 g every 12 hours for creatinine clearance (CL_Cr_) ≥ 50 ml/min, and 2 g every 24 hours or 36 hours for CL_Cr _< 50 ml/minute. Cefepime plasma concentrations were determined at several time-points before and after drug administration by high-pressure liquid chromatography. PK/PD parameters were computed by standard non-compartmental analysis.

**Results:**

Seventeen first-doses and 11 steady states (that is, four to six days after the first dose) were measured. Plasma levels varied greatly between individuals, from two- to three-fold at peak-concentrations to up to 40-fold at trough-concentrations. Nineteen out of 21 (90%) patients had PK/PD parameters comparable to literature values. Twenty-one of 21 (100%) patients had appropriate duration of cefepime concentrations above the MIC (T_>MIC _≥ 50%) for the pathogens recovered in this study (MIC ≤ 4 mg/l), but only 45 to 65% of them had appropriate coverage for potential pathogens with cefepime MIC ≥ 8 mg/l. Moreover, 2/21 (10%) patients with renal impairment (CL_Cr _< 30 ml/minute) demonstrated accumulation of cefepime in the plasma (trough concentrations of 20 to 30 mg/l) in spite of dosage adjustment. Both had symptoms compatible with non-convulsive epilepsy (confusion and muscle jerks) that were not attributed to cefepime-toxicity until plasma levels were disclosed to the caretakers and symptoms resolved promptly after drug arrest.

**Conclusions:**

These empirical results confirm the suspected risks of hidden side-effects and inappropriate PK/PD parameters (for pathogens with upper-limit MICs) in a population of ICU adult patients. Moreover, it identifies a safety and efficacy window for cefepime doses of 2 g every 12 hours in patients with a CL_Cr _≥ 50 ml/minute infected by pathogens with cefepime MICs ≤ 4 mg/l. On the other hand, prompt monitoring of cefepime plasma levels should be considered in case of lower CL_Cr _or greater MICs.

## Introduction

An empiric study in which the pharmacokinetics (PK) of imipenem were prospectively monitored in intensive care unit (ICU) children revealed wide inter-individual variations (up to four-fold at peak and >10-fold at through concentrations) that resulted in potentially too low dosages in 30% of cases [1]. Similar observations were also made with imipenem in adult patients [2,3], suggesting that drug adjustment algorithms used at the bedside might not be always accurate in unstable ICU patients, and that drug monitoring should be used more often [1].

The present report describes a similar quality assessment study in which the PK of cefepime was monitored in ICU adult patients. As in the children's study alluded to above [1], PK results were not disclosed to the caretakers unless clinical problems were suspected to be associated with inappropriate drug dosages. This observation is timely in light of two meta-analyses that reported an increased mortality (risk ratio 1.26 (95% CI 1.08 to 1.49)) in patients treated for severe infection with cefepime, as compared to patients treated with other beta-lactams [4,5]. Moreover, Bhat et al. [6] observed that bacteremia due to gram-negative pathogens with minimal inhibitory concentrations (MICs) of cefepime in the highest range of susceptibility (that is, 8 mg/l) or above [7] were associated with an increased mortality in patients treated with cefepime than in those treated with other antibacterials.

Alarmed by these reports, the Food and Drug Administration (FDA) completed a complementary meta-analysis of 88 comparative studies (including the 38 reported by Yahav et al) totalizing 9,467 cefepime-treated patients [8]. This analysis did not confirm a higher overall mortality related to cefepime. Nevertheless, in the absence of drug monitoring, the excess mortality or treatment failures reported in specific studies [4-6] could be related to untoward overdosing or underdosing of cefepime in unstable patients.

Ideal dosing of cefepime should obey pharmacokinetic/pharmacodynamic (PK/PD) population kinetics that help adjust drug dosing to the most appropriate PK/PD profile against target organisms [9-14]. This corresponds to a period of drug concentration above the MIC (T_>MIC_) of >40% to 60% for beta-lactams in general [15-20] and ≥50% for cefepime [19,20]. However, whether these goals are reached in the empiric day-to-day clinical setting is uncertain, especially in unstable ICU patients. The present work examined these issues in 21 consecutive ICU adult patients treated with cefepime. Individual PKs were prospectively determined following a similar study design as for imipenem in children [1]. The results further strengthen the need for antibiotic monitoring in complicated clinical situations.

## Materials and methods

### Experimental design

The Centre Hospitalier Universitaire Vaudois (CHUV) is a 1,400-bed tertiary teaching hospital encompassing all medical and surgical disciplines including organ grafts and burn patients. Its ICU is a mixed medico-surgical facility of 32 beds with a rate of admissions of approximately 2,600 patients per year. The study was aimed at following the natural PK profiles of cefepime in ICU adult patients, in a setting where beta-lactam monitoring was not routinely performed. It followed a similar protocol as in our former study of imipenem PK in the pediatric ICU [1]. In brief, all consecutive adult patients (≥18 years old) entering the ICU and prescribed cefepime (Bristol-Myers Squibb AG, Baar, Switzerland) by the caretakers were prospectively enrolled. All drug dosages and dosing-adjustments were decided by them, based on daily clinical and laboratory assessments. Patients were excluded if they were allergic to beta-lactams, had been treated with cefepime within the last 15 days, or were requiring dialysis at the time of inclusion. The results of cefepime monitoring were not disclosed to the caretakers until the end of the study, unless the caretakers or the principal investigators (TMC and PM) suspected clinical problems that might be associated with inappropriate drug concentrations [1]. The study aimed at collecting a total of 20 patients. The protocol was accepted by the local ethic committee, and written consent was obtained from the patient or from her or his legal representative.

Cefepime dosage in the ICU is 2 g every 12 h in patients with creatinine clearance (CL_Cr_) ≥50 ml/minute, and 2 g every 24 h or more in patients with CL_Cr _< 50 ml/minute. CL_Cr _was calculated by the Cockcroft-Gault equation [21]. CL_Cr _values reported herein are only those measured concomitantly to the determination of cefepime PKs. The drug was infused over 30 minutes via an intravenous line. PK analyses were performed at the first-dose and/or at steady state, that is, between Days 4 and 6 after treatment onset. Blood samples were drawn from a site remote from the drug administration line. In patients receiving the drug every 12 h, samples were collected just before drug administration, and at 30 minutes, 45 minutes, 1.5, 2.5, 4, 8 and 12 h after the beginning of drug infusion. In patients receiving the drug at longer intervals, in case of drug adaptation, blood sampling was made.

### Determination of cefepime concentrations in the plasma

Cefepime titration was performed as reported in a previous work [22]. Accordingly, to prevent ex-vivo drug degradation, blood samples were immediately chilled, centrifuged, and stored at -80°C until dosage was performed. All subsequent processes were performed at 4°C, including automatic injection by a refrigerated autosampler (Peltier cooler; Labsource, Reinach, Switzerland). Briefly, the procedure included initial extraction by protein precipitation, followed by reversed phase chromatography using 0.2 M Borate-Methanol (93%/7% vol/vol) mobile phase and integration of the 260 nm absorption signals. Calibration standards from 0.5 to 200 mg/l were prepared in healthy volunteer's plasma with cefepime provided by Bristol-Myers-Squibb AG (Sermoneta, Italy). Assay was carried out with a HPLC Merck-Hitachi LaChrom system (Hitachi Instruments, Ichige Hitachinaka, Japan)), and a LC_18 _150 × 4.6 mm column (Supelco, Bellefonte, PA, USA). More details on the method have already been published elsewhere [22]. Its limit of quantification is of 0.5 mg/l and the intra and inter run coefficients of variation are below or at 10.3%.

### PK parameters

Calculated PK parameters included the terminal slope of cefepime elimination from the plasma (*K*_β_), the area under the curve of cefepime plasma concentrations (AUC; 0 to 12 h), the area under the first moment curve (AUMC), the terminal half-life of cefepime in the plasma (*T*_1/2β _= log 2/*K*_*β*_), the mean resident time (MRT = AUMC/AUC), the systemic clearance (CL_CEF _= dose/AUC), and the initial and steady state volumes of distribution (*V*_β _= CL_CEF_/*K*_β _and *V*_ss _= CL_CEF _× MRT, respectively). For the seven-paired kinetics, comparisons between the first-dose PK and the steady-state PK were done by the Wilcoxon matched pairs test.

### Clinical and laboratory parameters, and PK/PD analyses

Characteristics of the patients are presented in Table 1. In addition, several clinical and biological variables were recorded daily during the ICU stay, including weight (using beds with weight assessment function), hemodynamic parameters (heart rate, mean blood pressure, central venous pressure), SAPS II score (Simplified Acute Physiology Score) [23], serum creatinine concentrations, creatinine clearance, urea, plasma proteins, serum albumin concentrations, blood lactate, pH, pCO_2_, HCO_3_, plasma sodium and potassium, aspartate aminotransferase (ALAT), alanine aminotransferase (ASAT), prothrombin time (PT), and hemoglobin. Throughout the PK determination period, hemodynamic parameters were recorded hourly for mean computation. Among clinical and laboratory parameters, those having a significant Pearson's correlation coefficient with any PK parameters were then selected for a stepwise multiple regression as predictive variable for the concerned PK parameters.

**Table 1:** Clinical and microbiological features of the study population (10 females and 11 males; median age 55.1 years, range 21.8 to 81.2)

Reason for ICU admission	Underlying disease	SAPS II score	Weight (Kg)	Cl_Cr_	Presumed pathogens	MIC (mg/l)
Cardiovascular surgery ^1^	Coronary artery disease	38	75	17.8		
Multiple trauma	Bipolar disorder	33	75	139.3	*E. coli*	0.024
Thoracic surgery ^2^	Non-specific interstitial pneumonia	33	85	126.6		
Abdominal surgery ^3^	Abdominal aortic aneurysms	26	75	51		
Multiple trauma	Chronic obstructive pulmonary disease	23	86	63.4	*S. aureus*	2
Abdominal surgery	Abdominal aortic aneurysms	32	85	32.9		
Cardiovascular surgery	Aortic stenosis	47	63	62.2		
Acute respiratory failure ^4^	Obesity stage II	24	120	135.5	*S. pneumoniae*	0.75
Neurosurgery	Cerebral arterio-venous malformation	50	53	166.9	*E. coli*	0.04
Cardiovascular surgery	Myeloproliferative disorder	52	65	79.6	*S. pneumoniae*	0.047
Multiple trauma	None	42	70	133.5	*P. aeruginosa*	4
Cardiovascular surgery	Aortic bicuspidy	9	68	101.9		
Acute respiratory failure ^1^	Coronary artery disease	51	60	12		
Neurosurgery	None	23	40	161	*P. aeruginosa*	4
Multiple trauma	Diabetes mellitus	22	58	92.1		
Cardiovascular surgery	Coronary artery disease	24	78	59.8		
Acute respiratory failure ^4^	Myeloproliferative disorder	69	52	95.5	*S. pneumoniae*	1
Cardiovascular surgery	Coronary artery disease	33	47	115.1		
Multiple trauma	None	24	62	142.1		
Ear-nose and throat surgery	Pharynx carcinoma	43	60	87.7		
Neurosurgery	High blood pressure	58	100	121.8	*H. influenzae*	1

Cl_Cr_, creatinine clearance at inclusion, as determined by the Cockcroft-Gault equation; *E. coli, Escherichia coli*; *H. influenza, Haemophilus influenza*; MIC, minimal inhibitory concentration; *P. aeruginosa, Pseudomonas aeruginosa*; *S. aureus, Staphylococcus aureus*;*S. pneumonia, Streptococcus pneumoniae*;

^1^Patients who developed drug accumulation and symptoms compatible with neurological toxicity.

^2^Patient suffering a further episode of bronchoaspiration; switched to amoxicillin-clavulanate during follow-up.

^3^Patient died eight days after leaving the ICU from multiorgan failure. Autopsy revealed an ischemic colitis with intra-abdominal steatonecrosis. Patient was also treated with metronidazole for the presence of *Clostridium difficile *in stool cultures.

^4^Patients eventually switched to levofloxacin as a treatment of choice of penicillin intermediate-resistant *Streptococcus pneumoniae*.

Presumed pathogens were identified at the central microbiology laboratory of the hospital and MICs of cefepime were determined by the E-test (AB Biodisk, Solna, Sweden). The T_>MIC _period is one of the most pertinent parameters predicting beta-lactam efficacy [15-20]. Therefore, this PK/PD parameter was computed for any kinetics provided by this study, using the cefepime MIC susceptibility breakpoints recommended by the Clinical and Laboratory Standards Institute (CLSI) (that is, ≤8 mg/l for *Enterobacteriaceae*, *Pseudomonas aeruginosa *and *Staphylococcus aureus*, ≤2 mg/l for *Haemophilus *spp. and ≤1 mg/l or lower for *Streptococcus pneumoniae *and other streptococci) [7].

### Evaluation endpoints

The primary endpoints were the appropriateness of the PK/PD profiles in terms of T_>MIC _regarding the recommended cefepime MIC breakpoints [7], as well as clinically-detected toxicity. The secondary endpoint was the fact that patients could be discharged from the ICU and eventually leave the hospital. On the other hand, treatment success was not a formal endpoint, as the study protocol was not designed to evaluate cefepime efficacy *strico sensu*. Cefepime was mostly used as first-line empiric treatment, and caretakers were free to switch to more standard therapy after receiving the results of microbial identification and susceptibility tests.

## Results

### Patient characteristics

Ten females and 11 males (median age 55.1 years, range 21.8 to 81.2) entered the study between 1 April and 30 September 2001. All consecutive eligible patients were included, and no patients were excluded after entry. Demographic details and laboratory features are presented in Table 1. Only patients with clinical and radiological features compatible with nosocomial pneumonia (as defined by onset of ≥48 h after hospitalization) were included. This bias toward nosocomial pneumonia is likely to result from the empiric nature of the study. Indeed, consecutive patients were included by the caretakers, who preferentially used cefepime monotherapy for empiric treatment of nosocomial pneumonia (we have notoriously few methicillin-resistant *Staphylococcus aureus *in our institution), while empirical treatment of other severe infections, mostly intra-abdominal, involves beta-lactams with anti-anaerobe activities (that is, penems or penams) sometimes combined with other drugs. Presumed bacterial pathogens cultured from bronchiolo-alveolar lavage were identified in 10/21 (47%) patients. They were all susceptible to cefepime according to the standard MIC cut-off values (Table 1) [7].

### Cefepime PK profiles

**Figure 1:**
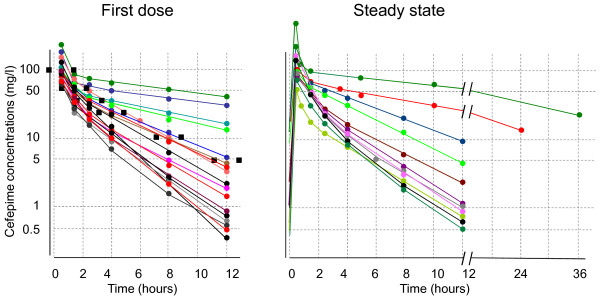
**Pharmacokinetic profile of cefepime**. Concentration of cefepime versus time determined in the plasmas of 21 consecutive patients as determined at the first dose (left panel; 17 individual PK profiles) or at steady state (right panel; 11 individual PK profiles). Colors identify individual patients.

Seventeen first-dose and 11 steady-state PK profiles were determined, among which both profiles were obtained in seven patients. Eleven patients had only first-dose PK determinations because they had already left the ICU by the time steady-state measurements should have been performed (that is, four to six days after treatment initiation). Conversely, four patients had only a steady-state measurement because they gave their written consent after the first dose had already been administered. The 12 h administration schedule was pursued in 19 patients and adapted in two patients with CL_Cr _<50 ml/minute (Figure 1). Figure 1 depicts the kinetics of cefepime concentrations in the plasma versus time at the first-dose (left panel) and at steady-state (right panel), respectively. Cefepime concentrations varied by two- to three-fold at peak levels and up to 40-fold at trough levels (Figure 1 and Table 2). The majority of patients (that is, 13/17 or 76% at first dose and 9/11 or 81% at steady state) had trough levels ≤10 mg/l. On the other hand, four patients clustered above this limit at the first dose, and two patients with altered renal function remained above this value at steady state, in spite of increasing the intervals of drug administration to 24 h and 36 h, respectively (right panel of Figure 1). These are the two patients who developed untoward neurological side effects.

**Table 2:** Pharmacokinetic parameters and comparison with previous literature using cefepime dosage of 2 g q 12 h.

Parameters and time of calculation	Mean reported values ± SD			
	
	Present study	**Barbhaiya et al. ^3 ^**[45]	**Sampol et al.^4 ^**[50]	**Bonapace et al.^4 ^**[44]
First dose (17 patients)				
T_1/2β _(h)	4.03 ± 3.19	NS	2.45 ± 0.56	2.8 ± 0.6
C_Max _(mg/l) ^1^	105 ± 22	132 ± 21	NS	102 ± 15 ^5^
C_Min _(mg/l)^1^	7.6 ± 12	NS	NS	NS
AUC (mg.h/liter)	370 ± 360	268 ± 27	217 ± 34	224 ± 59
MRT (h)	5.1 ± 4.64	2.56 ± 0.31	NS	NS
Clearance (liter/h.kg) ^1,2^	0.130 ± 0.077	NS	0.152 ± 0.025	0.1 ± 0.03
V_β _(liter/kg)	0.513 ± 0.180	NS	NS	NS
V_SS _(liter/kg)	0.413 ± 0.118	NS	0.36 ± 0.1	0.43 ± 0.1
Steady state (11 patients)				
T_1/2β _(h)	4.33 ± 4.32	Not available	2.62 ± 0.53	Not available
C_Max _(mg/l) ^1^	97 ± 8		NS	
C_Min _(mg/l)^1^	2.68 ± 3.06		NS	
AUC (mg.h/liter)^1^	226 ± 107		262 ± 57	
MRT (h)	5.3 ± 5.9		NS	
Clearance (liter/h.kg)	0.131 ± 0.084		0.133 ± 0.029	
V_β _(liter/kg)	0.513 ± 0.180		NS	
V_SS _(liter/kg)	0.413 ± 0.118		0.35 ± 0.1	

C_Max _and C_MIN_, maximal and minimal plasma concentrations at the end of drug infusion and just before the next infusion, respectively; AUC, area under the curve; MRT, mean residence time; NS, not specified; T_1/2β_, terminal plasma half-life; V_β_, initial volume of distribution; V_SS_, volume of distribution at steady state

^1^only patients with 2 g q 12 h (without two cases with dose adjustment at steady state)

^2^extrapolated to infinity for the first PK

^3^in normal volunteers

^4^in burn patients

PK parameters were stable in most patients, with the notorious exception of the two patients with altered renal function (CL_Cr _= 19 and 12 ml/minute, respectively). Table 2 shows that patients with conserved renal function (that is, a CL_Cr _≥50 ml/minute) had relatively comparable PK parameters as compared to those previously reported in healthy volunteers or burn patients. The main difference in our cohort was a greater T_1/2β _(h) and a parallel increased mean residence time (MRT).

### Factors influencing PK profiles

**Figure 2:**
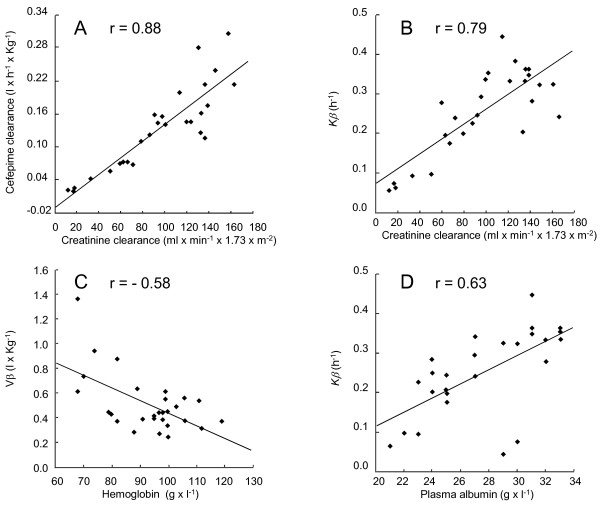
**Significant correlations between physiological and pharmacokinetic parameters**. Cefepime elimination closely correlated with creatinine clearance (panels **A **and **B**), as abundantly described [15-20]. In addition, more intricate parameters also showed independent negative and positive correlations with drug elimination, as for instance the concentrations of hemoglobin (panel **C**) and plasma albumin (panel **D**). Corresponding coefficients of correlations (r values) are indicated. Additional correlations are presented in Table 3.

To further dwell on factors influencing cefepime kinetics we attempted to match clinical and laboratory co-variables with specific PK parameters. Some associations were straightforward, such as the direct correlation between Cl_Cr _and the steepness of the slope of elimination of cefepime from the plasma (that is, the terminal slope of cefepime clearance, or *K*_β_, which follows the steeper slope of initial rapid drug distribution, or *K*_α_) (Figure 2A, B), and between hemodilution and volume of distribution (*V*_β_) (Figure 2C). These are also the parameters most likely to be taken into account for drug dosing adjustment by clinicians.

Table 3 presents some of these parameters. Although several are easily associated with hemodynamic conditions, others could be more intricately involved in drug elimination, as exemplified by the reported pH-dependent, plasma-dependent, and temperature-dependent degradation of cefepime [22,24,25]. In this line, both the pCO_2 _and the HCO_3 _were significantly associated with decreased drug half-life and mean resident time. Thus, in complex clinical situations the PK profiles might be influenced by individual physiopathological variables that are not taken into account in standard algorithms for adjustment of drug dosages.

**Table 3:** Combined two-by-two correlations and multiple regression between clinical and laboratory parameters, and PK values.

Clinical and laboratory parameters		Pharmacokinetic parameters ^1,2^(number of data points)				
		
	*K*_β_	*T*_1/2β_	MRT_iv_	CL_CEF_	*V*_*β*_	*V*_SS_
**Weight**	0.08	-0.05	-0.04	-0.34	-0.42**	-0.47**
	(28)	(28)	(28)	(28)	(28)	(28)
**Age**	-0.65**	0.58*	0.61*	-0.75**	-0.50**	-0.34
	(28)	(28)	(28)	(28)	***(28)***	(28)
**Proteins**	0.52*	-0.27	-0.28	0.24	-0.13	-0.29
	(26)	(26)	(26)	(26)	(26)	(26)
**Albumin**	0.63*	-0.31	-0.32	0.21	-0.27	-0.43**
	***(26)***	(26)	(26)	(26)	(26)	(26)
**Hemoglobin**	0.07	0.06	0.07	-0.42**	-0.58**	-0.59**
	(28)	(28)	(28)	(28)	***(28)***	***(28)***
**Na^+^**	-0.11	0.01	-0.01	0.17	0.38	0.38
	(28)	(28)	(28)	(28)	(28)	(28)
**Creatinine**	-0.78**	0.91*	0.91*	-0.69**	-0.31	-0.19
	(27)	(27)	(27)	(27)	(27)	(27)
**CL_Cr_**	0.79*	-0.81**	-0.82**	0.88*	0.51*	0.35
	***(27)***	***(27)***	***(27)***	***(27)***	(27)	(27)
**pCO_2_**	0.28	-0.41	-0.42**	0.03	-0.14	-0.20
	(23)	(23)	(23)	(23)	(23)	(23)
**HCO_3_**	0.33	-0.42**	-0.41	-0.05	-0.27	-0.33
	(23)	(23)	(23)	(23)	(23)	(23)
**Cefepime dose (mg/kg)**	0.19	-0.17	-0.17	0.57*	0.51*	0.53*
	(28)	(28)	(28)	***(28)***	(28)	(28)

^1^Significant Pearson's coefficients with *P *< 0.05 are highlighted by asterisk. One asterisk indicates positive (direct) correlations and two asterisk indicate negative (inverse) correlations.

^2^For each PK parameters, the most pertinent physiological parameters according to the result of the two by two correlations were included as independent variable in a forward stepwise multiple regression. Creatinine serum levels were excluded from the analysis (in spite of a significant correlation with some pK parameters) because of a non-normal (bimodal) distribution. Creatinine clearance, which shares similar biological information, was more regularly distributed. Remaining primary predictive variable (*P *< 0.05) after this procedure are marked in bold italic font in the table.

### Side effects

The protocol was not aimed at detecting specific side effects of cefepime therapy. Therefore, possible related side effects were left on the appreciation of the caretakers, based on daily complete clinical and laboratory assessments. No untoward side effects were attributed to cefepime by the caretakers at first. Yet the two (10%) patients with high concentrations of cefepime in the plasma (highest concentrations in right panel in Figure 1) presented episodes of confusion and flapping tremor compatible with metabolic encephalopathy. Both had altered renal functions and had been subjected to dosing adjustment (2 g of cefepime q 24 h and 36 h for the patients with CL_Cr _of 19 and 12 ml/minute, respectively). Yet, this dosage adjustment was insufficient and they had nevertheless high plasma levels. The accumulation of cefepime in the plasma concentrations was disclosed to the medical staff, and both patients recovered within 24 h of treatment arrest.

### Pharmacodynamic profiles and clinical outcome

Optimal beta-lactam efficacy requires T_>MIC _of >60% to 70% for *Enterobacteriaceae *and streptococci, and 40 to 50% for *Staphylococcus aureus *[15-19,26]. For certain beta-lactams including cefepime, a lower limit of 50% was also suggested [19,20]. Table 4 presents the T_>MIC _of the present patient population as determined for cefepime MICs of 4 and 8 mg/l, respectively. At the dosage used herein (that is, 2 g q 12 h in patients with CL_Cr _≥50 ml/minute) all patients had T_>MIC _values above 50% for cefepime MIC of ≤ 4 mg/ml. Thus, the theoretical PD coverage was appropriate for all the presumed pathogens recovered in this study (cefepime MIC ≤4 mg/l). All patients in this study were discharged from the ICU without antibiotic treatment failure regarding the indication of cefepime treatment, and all except one (Table 1) could eventually leave the hospital. On the other hand, when increasing the cefepime MIC cut-off to 8 mg/ml, T_>MIC _decreased to ≤67% at the first dose and <44% at steady state, indicating that the dosage would be inadequate in a substantial number of patients infected with Gram-negative pathogens with such borderline susceptibilities, as suggested by Bhat et al. [6].

**Table 4:** Time over MIC (T_>MIC_) of total cefepime in patients without renal failure (CL_Cr _> 50 ml/minute)

	1st dose (N patients = 15)		steady state (N patients = 9)	
	
T_>MIC_	≤4 mg/ml ^1^	8 mg/ml ^1^	≤4 mg/ml ^1^	8 mg/ml ^1^
>0.3 (3:36 h)	100%	100%	100%	100%
>0.4 (4:48 h)	100%	87%	100%	67%
>0.5 (6:00 h)	100%	67%	100%	44%
>0.6 (7:12 h)	67%	47%	67%	22%
>0.7 (8:24 h)	53%	40%	33%	22%

^1^Cefepime MIC

## Discussion

The present empirical study confirms the great inter-individual variability of cefepime PK in the clinical setting, as reported with cefepime and imipenem by others [1,2,27,28]. Moreover, it underlines the difficulty of bedside prediction of cefepime PK, based on standard drug adjustment algorithms, including calculated CL_Cr_. In the present series, this resulted in extreme cefepime concentrations in the plasma from rather low values (trough cefepime concentrations below 4 mg/l in ca 50% of the patients) (Figure 1) to unpredicted toxic values in two other patients with renal impairment.

A major parameter for cefepime drug adjustment is CL_Cr_, which is often calculated by the classical Cockcroft-Gault equation [21]. However, calculated clearance may be subject to errors because it does not take into account features such as muscular mass and turnover, which may influence creatinine concentrations in the serum [29]. Therefore, biases in calculated CL_Cr _could be one potential explanation for the inter-individual PK variability observed. Nevertheless, although the Cockcroft-Gault equation may suffer from inaccuracies, the calculated CL_Cr _values correlated very well with cefepime clearance, as indicated in Figure 2. Additionally, we also tentatively calculated CL_Cr _values using the MDRD (Modification of Diet in Renal Disease) method [30], but the results were quite concordant with the values presented herein (data not presented). Hence, some of the variations might be due to other factors.

For instance, some patients had increased CL_Cr _as previously reported (>120 ml/minute, Figure 2) [31] and might have benefited from increasing drug dosages. Alternatively, additional more intricate parameters presented in Table 3 might also interfere. Among these, some relations were expected, such as the direct correlation between Cl_Cr _and cefepime elimination, whereas others were less obvious, such as the direct correlation between the concentration of plasma albumin and *K*_β _(Figure 2D). Depending on the circumstances, high plasma albumin may be associated either with dehydration, which could result in poor renal perfusion and decreased cefepime clearance, or with good cardiovascular performance and good cefepime clearance, which was likely to be the case herein.

Other parameters for initial dosing are weight and gender, which might call less attention by the caretakers in adult than in pediatric medicine. However extreme weights in our series varied by three times (Table 1) and were not likely to explain the up to 40-times difference in drug levels observed. Moreover, similar variations were observed in other PK studies [2,3], and especially in children, where weight is a prime consideration in drug dosing decision [1]. Taken together, the extreme variations observed are likely to result from intricate interactions between multiple factors, which are by no way simple to integrate in the bedside decision process.

Most patients with a preserved renal function had stable individual PK profiles over time in spite of a wide range of CL_Cr _values ranging from 160 to 53 ml/minute (Figure 2), and the fact that no drug adjustments were performed. In contrast, drug accumulation and toxicity was observed in two patients with renal impairment (CL_Cr _< 50 ml/minute), in spite of drug adjustment. This is potentially important because caretakers did not attribute neuropsychological alterations, which may be multi-factorial in ICU conditions, to drug toxicity until the high concentrations of cefepime were disclosed to them and the symptoms resolved promptly after treatment arrest. Moreover, there is a lack of information in the literature regarding the threshold of cefepime plasma levels predicting neurotoxicity. Indeed, out of 35 patients with cefepime-induced neurological complications reported in 10 studies (excluding reviews and chronic dialysis patients) [27,28,32-39], the concentrations of cefepime were determined in only one case (in the plasma and the CSF) and were quite high, that is, 284 mg/l and 18 mg/l, respectively [28]. Besides, only one recent study in neutropenic patients with mild renal failure indicated that trough plasma concentrations of cefepime above 22 mg/l were likely to be associated with encephalopathy [40]. The main constant over all the reported cases is the association of neurotoxicity with renal impairment. While renal impairment implies possible drug accumulation, it might also potentiate the effect of additional neurotoxic factors, including factors related to the patient, or maybe the C-3' substituent *N*-methylpyrrolidine metabolite of cefepime, which may accumulate in the case of renal failure [25,41]. Thus, the threshold of toxicity might be patient-dependent. On the other hand, most studies examining the PK produced by 2 g of the drug administered intravenously or intramuscularly to healthy volunteers or patients without renal failure report trough cefepime concentrations in the plasma ≤10 mg/l in [9,11,42-46], which was also the case herein. Therefore a safe assumption is that trough concentrations of >10 mg/l of cefepime should alert the clinician on the risk of neurotoxicity in susceptible patients, and concentrations of >20 mg/l should probably be avoided.

On the other extreme, too low dosages may result in treatment failures, at least as predicted by PK/PD studies [15-19,26]. Postulating that T_>MIC _measured is pertinent to predict clinical outcome, then all of our patients had appropriate coverage of cefepime (T_>MIC _≥ 50%) as recently proposed [19,20] for the presumed bacterial pathogens recovered herein (MIC ≤ 4 mg/l) (Table 4). On the other hand, if one postulates an MIC of 8 mg/l, which was associated with treatment failures in patients with bacteremia due to Gram negative pathogens [6], then close to 50% of the patients would have had an inappropriate coverage (T_>MIC _> 50%). This is of particular concern when considering problematic pathogens such as those producing extended-spectrum beta-lactamases, or *P. aeruginosa *and *Acinetobacter *spp., which may have high cefepime MICs (≥8 mg/l) and pose major therapeutic challenges, and if one takes into account that up to 20% of the total drug is bound to serum proteins [47,48]. Moreover, in addition to pure MIC concerns, a recent study identified *P. aeruginosa *infection, mechanical ventilation, and neutropenia as independent risk factors for cefepime treatment failure [49]. Higher cefepime doses were proposed to overcome some of these issues (for example, 2 g q 8 h) [9], but high doses may also increase the risk of neurological side effects. Hence, adjusting dosage on the basis of drug monitoring is reasonable in such cases.

## Conclusions

Taken together, these results of drug monitoring independently validate the population kinetics of cefepime elaborated by others [9-14]. Moreover, they show that empirical drug dosing following standard drug adjustment algorithms in the ICU is not accurate enough to prevent extreme PK deviations, which might be one or the possible explanations for the toxicity and treatment failure problems reported by Yahav et al. [4] and Bhat et al. [6]. Eventually, they indicate that 2 g of cefepime q 12 h is safe and effective for patients with CL_Cr _≥ 50 ml/minute and against pathogens with cefepime MICs ≤ 4 mg/l, but that drug monitoring should be considered in any conditions falling outside these limits.

## Key messages

• 2 g of cefepime every 12 h was safe and appropriate for patients with CL_Cr _≥50 ml/min pathogens with cefepime MICs ≤4 mg/l.

• However, this dosage was too low up to 50% of more of patients infected with microbes with greater cefepime MICs (≥8 mg/l).

• Moreover, cefepime accumulation and neurological toxicity (non-convulsive epilepsy) occurred in two patients with CL_Cr _<50 ml/minute, in spite of drug dosage adjustment.

• Monitoring of cefepime plasma levels is warranted in patients with CL_Cr _<50 ml/minute and infection due to pathogens with cefepime MICs ≥8 mg/l.

## Abbreviations

AUC: area under the curve; AUMC: area under the first moment curve; CL_Cr_: creatinine clearance; CLSI: Clinical and Laboratory Standards Institute; FDA: Food and Drug Administration; HPLC: high pressure liquid chromatography; ICU: intensive care unit; MIC: minimal inhibitory concentration; MDRD: modification of diet in renal disease; MRT: mean resident time; PD: pharmacodynamics; PK: pharmacokinetics; SAPS II: simplified acute physiology score; V_β_: volume of distribution.

## Competing interests

The authors declare that they have no competing interests.

## Authors' contributions

TMC collected the data. TMC, EG, DB and PM initiated the study, and the design. TMC, DB and PM were involved in the interpretation of the results. TMC wrote the manuscript, DB and PM helped to draft the manuscript. EG, PAM, RC, MDS, MMB and LD contributed to the conception of the study and revision of the manuscript. PM and DB provided the final revision of the manuscript. SB provided technical support for the study. All authors read and approved the final manuscript.
